# Spatiotemporal roles of AMPK in PARP-1- and autophagy-dependent retinal pigment epithelial cell death caused by UVA

**DOI:** 10.1186/s12929-023-00978-4

**Published:** 2023-11-07

**Authors:** Anthony Yan-Tang Wu, Ponarulselvam Sekar, Duen-Yi Huang, Shu-Hao Hsu, Chi-Ming Chan, Wan-Wan Lin

**Affiliations:** 1 Department of Pharmacology, College of Medicine, National Taiwan University, Taipei, Taiwan; 2 Chemical Biology and Molecular Biophysics Program, Taiwan International Graduate Program, Academia Sinica, Taipei, Taiwan; 3 Graduate Institute of Medical Sciences, Taipei Medical University, Taipei, Taiwan; 4 Department of Ophthalmology, Cardinal Tien Hospital, New Taipei City, Taiwan; 5 School of Medicine, Fu Jen Catholic University, New Taipei City, Taiwan

**Keywords:** RPE, UVA, ROS, AMPK, PARP, Autophagic cell death, Lysosome dysfunction, EGFR

## Abstract

**Background:**

Although stimulating autophagy caused by UV has been widely demonstrated in skin cells to exert cell protection, it remains unknown the cellular events in UVA-treated retinal pigment epithelial (RPE) cells.

**Methods:**

Human ARPE-19 cells were used to measure cell viability, mitochondrial reactive oxygen species (ROS), mitochondrial membrane potential (MMP), mitochondrial mass and lysosomal mass by flow cytometry. Mitochondrial oxygen consumption rate (OCR) was recorded using Seahorse XF flux analyzer. Confocal microscopic images were performed to indicate the mitochondrial dynamics, LC3 level, and AMPK translocation after UVA irradiation.

**Results:**

We confirmed mitochondrial ROS production and DNA damage are two major features caused by UVA. We found the cell death is prevented by autophagy inhibitor 3-methyladenine and gene silencing of ATG5, and UVA induces ROS-dependent LC3II expression, LC3 punctate and TFEB expression, suggesting the autophagic death in the UVA-stressed RPE cells. Although PARP-1 inhibitor olaparib increases DNA damage, ROS production, and cell death, it also blocks AMPK activation caused by UVA. Interestingly we found a dramatic nuclear export of AMPK upon UVA irradiation which is blocked by N-acetylcysteine and olaparib. In addition, UVA exposure gradually decreases lysosomal mass and inhibits cathepsin B activity at late phase due to lysosomal dysfunction. Nevertheless, cathepsin B inhibitor, CA-074Me, reverses the death extent, suggesting the contribution of cathepsin B in the death pathway. When examining the role of EGFR in cellular events caused by UVA, we found that UVA can rapidly transactivate EGFR, and treatment with EGFR TKIs (gefitinib and afatinib) enhances the cell death accompanied by the increased LC3II formation, ROS production, loss of MMP and mass of mitochondria and lysosomes. Although AMPK activation by ROS-PARP-1 mediates autophagic cell death, we surprisingly found that pretreatment of cells with AMPK activators (A769662 and metformin) reverses cell death. Concomitantly, both agents block UVA-induced mitochondrial ROS production, autophagic flux, and mitochondrial fission without changing the inhibition of cathepsin B.

**Conclusion:**

UVA exposure rapidly induces ROS-PARP-1-AMPK-autophagic flux and late lysosomal dysfunction. Pre-inducing AMPK activation can prevent cellular events caused by UVA and provide a new protective strategy in photo-oxidative stress and photo-retinopathy.

## Introduction

UV radiation-induced photochemical damage can promote aging and decrease longevity in multiple organs including the skin and the eyes (e.g., cornea and retina). Oxidative stress is one of the major cellular events resulting from UV radiation which has been reported to accelerate skin aging owing to the accumulation of reactive oxygen species (ROS) [1]. UV can be subcategorized into 3 different wavelengths which, from the lowest to the highest energy, are UVA (315–400 nm), UVB (280–315 nm), and UVC (100–280 nm). Even though the anterior structures of the eye such as the cornea and the lens are able to absorb and block UVB and UVC, UVA, which accounts for about 95% of solar UV radiation, can still penetrate and reach the retina. Retinal pigment epithelium (RPE) located between the photoreceptors and the choroid is one of the major sites to arise oxidative stress in the eyes and is involved in UV-induced phototoxicity [2]. In young subjects, RPE cells can mitigate ROS generation by increasing antioxidant defenses whereas such antioxidant mechanism is impaired thus causing retinal cell death in the elderly [3].

Autophagy is an evolutionally conserved catabolic cellular process. Accumulated evidence indicates that ROS production can mediate autophagy formation, which then provides protection against oxidative stress by clearing the damaged proteins, lipids, and DNA, and restoring metabolic homeostasis [4, 5]. On the other hand, excess autophagy leads to cell death and has been shown to be involved in rheumatic diseases [6], diabetic kidney disease [7], cardiac disease [8] and glaucoma [9]. In this aspect, UV radiation-dependent genotoxic stress induces autophagy and upregulation of autophagy markers [10–12]. The underlying mechanisms include AMPK activation [10] and p53-dependent gene transcription of AMPK and Sestrin 1/2 [13, 14]. In contrast, UV-induced skin photoaging and pigmentation are partially resulting from activating the PI3K/AKT/mTOR pathway and then inhibiting autophagy [15, 16]. Moreover, studies have revealed that UV-induced autophagy is the consequence of oxidative stress-mediated DNA damage [17, 18]. UV-induced damages in dermal fibroblasts [19, 20], keratinocytes [21], and skin [15, 22] are protected by the upregulated autophagy, suggesting that autophagy serves a pro-survival role in the skin. One possible underlying mechanism for the survival role of autophagy is its ability to help DNA damage repair upon UV radiation [23, 24]. In RPE cells, constitutive autophagy also plays a critical role in maintaining cell function and normal vision [25, 26].

To date, a vast majority of the studies using UV radiation to elucidate the molecular events is most recognized in skin [12, 24, 27–30] and is partially associated with EGFR. EGFR plays an important role in the development and normal physiology of epithelial cells and keratinocytes, such as stimulating cell proliferation, differentiation, and migration. Similarly, EGFR induces proliferation, differentiation and migration of RPE cells, contributing to proliferative vitreoretinopathy and blindness [31–34]. Even though EGFR transactivation can be induced by UV irradiation in keratinocytes [35], it remains unknown of the effect of UV radiation on autophagy in retinal cells, not to mention its pro-survival or pro-death role, the underlying signaling cascade and interplay with DNA damage repair.

Therefore, in this study, we explored the death mode and underlying mechanisms resulting from oxidative stress like autophagy, DNA damage, AMPK, EGFR, and lysosomal dysfunction in UVA-irradiated RPE cells. As a result, we found UVA causes autophagy associated cell death in ARPE-19 cells *via* rapid induction of mitochondrial ROS-PARP-1-AMPK-autophagic flux axis which is accompanied by a late and ROS-AMPK-independent death pathway mediated by cathepsin B. Of note, pre-treatment of RPE cells with AMPK activators A769662 and metformin can rescue cells by maintaining mitochondrial dynamics and inhibiting mitochondrial ROS production and DNA damage, without affecting lysosomal dysfunction. In contrast, treatment with EGFR tyrosine kinase inhibitors (TKIs) enhances UVA-induced cell death *via* increasing autophagic flux and lysosomal dysfunction.

## Materials and methods

### Reagents

Metformin, mitoTEMPO, N-acetyl cysteine (NAC), 3-methyladenine (3-MA), bafilomycin A1 (BafA1), necrostatin-1, oligomycin, carbonyl cyanide-p-trifluoromethoxyphenylhydrazone (FCCP), and rotenone were from Sigma-Aldrich (St. Louis, MO, USA). Gefitinib and olaparib were purchased from SelleckChem (Houston, TX, USA). Afatinib was purchased from AddoQ BioScience (Irvine, CA, USA). CA-074Me and antimycin A were purchased from Merck Millipore (Massachusetts, USA). Dihydroethidium (DHE), MitoSOX Red, MitoTracker green, LysoTracker Red, DMEM/F12, trypsin–EDTA, penicillin, streptomycin, and amphotericin B were from Invitrogen (Waltham, MA, USA). A769662, zVAD-FMK and mitoTEMPO were from MedChemExpress (Deer Park, NJ, USA). Antibodies against p62/SQSTM1, Drp-1, PARP-1, TOM20 and phosphorylated forms of AMPKα (T172), Drp-1 (S616) and EGFR (Y1068) were from Cell Signaling Technology (Danvers, MA, USA). Antibodies against AMPKα and β-actin were from Santa Cruz Biotechnology (Dallas, TX, USA). EGFR antibody was from Merck Millipore. PAR antibody was from Trevigen (Gaithersburg, MD, USA). LC3 antibody was from Genetex (Irvine, CA, USA). LAMP1 antibody was from Abcam (Cambridge, UK). TRPML1 antibody was from Sigma-Aldrich. ATP6V0D1 antibody was from Proteintech (Rosemont, IL, USA). The ECL reagent (Western blotting lightening chemiluminescence reagent plus) was purchased from PerkinElmer (Waltham, MA, USA).

### Cell culture

Adult human RPE cell line ARPE-19 was purchased from the Food Industry Research and Development Institute (Hsinchu, Taiwan). These cells were maintained in DMEM/F12 supplemented with 10% fetal bovine serum (Gibco, Carlsbad, CA, USA), 100 units/ml penicillin, 100 μg/ml streptomycin, and 25 µg/ml amphotericin B. The cells were cultured in a humidified incubator at 37 °C and 5% CO_2_. For all the experiments prior to UVA irradiation, cells reaching 70–80% of confluence were starved and synchronized in serum-free DMEM for 12–16 h before they were subjected to experiments. In most experiments, UVA at 12.6 J/cm^2^ was applied unless otherwise specified.

### siRNA transfection

Human siATG5 (Cat no. EHU085781) and scramble nonspecific siRNA were purchased from Sigma-Aldrich. ARPE-19 cells at 60% confluence were transfected with 100 nM siRNA by DharmaFECT Transfection Reagents (Horizon Discovery, Waterbeach, UK) following the manufacturer’s instruction. At 48 h post transfection, cells were irradiated with UVA and then harvested for analysis.

### Cell viability assay

The cell viability analyses were assessed using Annexin V-FITC Apoptosis Detection Kit with PI (Biolegend, San Diego, CA, USA). Briefly, 16–18 h post-UVA irradiation, cells with different pretreatments were washed with PBS and re-suspended in 0.2 ml cold binding buffer. Then, 1 μl of Annexin V-FITC and 2 ul of propidium iodide (PI) were added and the cells were incubated for 30 min in a humidified incubator at 37 °C and 5% CO_2_. Following incubation, the cells were centrifuged at 300 × g for 5 min and the supernatant was removed. The cell pellets were re-suspended in 0.5 ml cold binding buffer. Cell samples were placed on ice, away from light, and FITC and PI fluorescences were immediately measured by using flow cytometer (FACSCalibur, Becton, Dickinson and Company, Franklin Lakes, NJ, USA). Data were analyzed using Cell Quest Pro software (Becton, Dickinson, and Company).

### Flow cytometric measurements of cytosolic ROS, mitochondrial ROS, mitochondrial membrane potential, mitochondrial and lysosomal mass

ARPE-19 cells with indicated pretreatment were subjected to UVA (12.6 J/cm^2^) irradiation. At the indicated time points, cells were harvested and sent to flow cytometer (FACSCalibur, Becton, Dickinson and Company) for analysis. Dihydroethidium (DHE) and its mitochondrion-targeted form mitoSOX (each of 5 μM) were used to detect cellular and mitochondrial superoxide (O_2_^−^). Mitochondrial mass was measured by Mitotracker green (200 nM). Mitochondrial membrane potential was measured by JC-1 dye (2 μM). Lysosomal mass was measured by LysoTracker Red DND-99 (75 nM). All the fluorescence signals were detected using flow cytometry (FACS Calibur system Franklin Lakes, NJ, USA) and represented as percentages to the control group.

### Immunocytochemistry

ARPE-19 cells with different pretreatments were harvested at 1 h post-UVA irradiation. For the Mitotracker red CRXROS (Cell Signaling) staining, Mitotracker was added to the cells with a final concentration of 100 nM at 30 min prior to paraformaldehyde fixation. All groups were initially fixed with 4% paraformaldehyde at 37 °C followed by permeabilization with 0.2% Triton X-100 for 15 min, and blocking by BSA (5%) in TBS for 1 h. For mitochondrial morphology and LC3 status observation, immunostaining was then performed using primary antibody against TOM20 (1:500) and/or LC3 (1:500) in 1% BSA overnight at 4 °C. For the AMPK translocation, the primary antibody against AMPK (1:500) in 1% BSA was used for incubation overnight at 4 °C. After washing with TBS 3 times, cells were incubated with secondary antibody in 1% BSA in TBS for 1 h at room temperature and then mounted with DAPI Fluoromount-G (SouthernBiotech, Birmingham, AL, USA). Images were acquired using a 100 X Plan-Neofluar oil objective of LSM 780 microscopy (Carl Zeiss, Jena, Germany).

### Immunoblotting

Post-UVA irradiation, the medium from 12-well cell culture plates was aspirated and the cells were rinsed twice with ice-cold PBS and 80 μl of sodium dodecyl-sulfate polyacrylamide gel electrophoresis (SDS-PAGE) 1X sample loading buffer (diluted with radioimmunoprecipitation assay buffer) was then added to each well. After harvesting, cell lysates were sonicated and heated at 98 °C for 10 min followed by centrifuging with 10,000 × *g* at 4 °C for 1 min. SDS-PAGE was performed and transferred to a polyvinylidene difluoride membrane. Non-specific binding was blocked with TBST (50 mM Tris–HCl, pH 7.5, 150 mM NaCl, and 0.02% Tween 20) containing 5% non-fat milk for 1 h at room temperature. After immunoblotting with the first specific antibody at 4 °C overnight, membranes were washed three times with TBST and incubated with a horseradish peroxidase (HRP) conjugated secondary antibody for 1 h. The dilution folds of the first specific antibodies and β-actin were 1:1000 and 1:10,000, respectively. After three washes with TBST, the protein bands were detected with an enhanced chemiluminescence detection reagent. To make sure equal amounts of sample protein were applied for electrophoresis and immunoblotting, β-actin was used as an internal control.

### Quantitative real-time polymerase chain reaction (qRT-PCR)

Total RNAs were isolated from UVA-irradiated ARPE-19 cells using Qiazol (QIAGEN, Hilden, Germany) and subjected to qRT-PCR to quantify mRNA expressions of human transcription factor EB (TFEB). Total RNAs were first reversely transcribed into complementary DNA using SuperScript™ IV First-Strand Synthesis System (Invitrogen) followed by qPCR with SYBR Green PCR Master Mix (Invitrogen) using a StepOnePlus Real-Time PCR System (Applied Biosystem, Waltham, MA, USA, at 95 °C for 10 min, 40 cycles at 95 °C for 15 s, and 62 °C for 1 min. The relative expressions of genes were calculated using ΔΔCT method. Primer sequences against hTFEB were: 5′-TGGCAACAGTGCTCCCAATA-3′ (TFEB-forward) and 5′-GTACACATTCAGGTGGCTGCT-3′ (TFEB-reverse); hp62/SQSTM1 were 5′-GCCAGAGGAACAGATGGAGT-3′ (hp62/SQSTM1-forward) and 5′- TCCGATTCTGGCATCTGTAG-3′ (hp62/SQSTM1-reverse); and hLC3 were 5′- GAGAGCAGCATCCAACCAAA-3′ (hLC3-forward) and 5′- ACATGGTCAGGTACAAGGAAC-3′ (hLC3-reverse).

### Mitochondrial oxygen consumption rate

The oxygen consumption rate (OCR) was measured by the extracellular flux analyzer XF24 (Seahorse Bioscience, Houston, TX, USA). ARPE-19 cells were plated at 4 × 10^5^ cells/well in a Seahorse 24-well V7 microplate (Seahorse Bioscience) and cultured in DMEM/F12 for 24 h in a 5% CO_2_ incubator at 37 °C. Then, the medium was removed and cells were incubated in XF assay medium in the absence of FBS for 1 h at 37 °C in a measuring chamber without CO_2_ input. The mitochondrial complex inhibitors (oligomycin, FCCP, rotenone, and antimycin A) were freshly prepared in XF assay media. A769662 (25 μM) or metformin (6 mM) was added into wells 30 min prior to UVA irradiation. At 1 h post-UVA irradiation, the plate was subjected to the Seahorse XF24 extracellular flux analyzer. After 26 min of measuring the basal respiration, oligomycin (2.5 μM) was injected followed by FCCP (1 μM) at 50 min, rotenone (2.5 μM) and antimycin A (2.5 μM) at 74 min. OCR was recorded as pMoles per minute, and calculated as percentage of the OCR value before the treatment of tested agents. ATP turnover and respiratory capacity were measured and calculated after the sequential treatments with oligomycin and FCCP as previously described [36]. Averages of three wells were taken per data point. Antimycin A is an inhibitor of ATP synthase, so OCR reduction after antimycin A treatment represents ATP turnover under specific conditions. FCCP is an uncoupling agent of electron transport and can generate a proton efflux to induce the maximum respiration termed respiratory capacity or uncoupled respiration.

### Intracellular cathepsin B activity assay

Intracellular active cathepsin B released from the destabilized lysosomes was determined by the Magic Red^TM^ cathepsin detection kit (Part #937; ImmunoChemistry Technologies, Bloomington, MN, USA). After UVA-irradiation, ARPE-19 cells were harvested at indicated time points then centrifuged at 300 × *g* for 5 min, re-suspended in 1 ml fresh medium and treated with cathepsin B substrate, which was reconstituted with ddH_2_O to make a reagent solution in the ratio of 1:10. After incubating at 37 °C for 30 min, cells were washed twice with PBS and re-suspended with 0.5 ml of PBS then immediately measured by using flow cytometer (FACSCalibur, Becton, Dickinson and Company, Franklin Lakes, NJ, USA).

### Statistical analysis

Data were expressed as mean ± S.E.M. Multiple groups were compared by one-way analysis of variance followed by Bonferroni or Tuckey post-test, making use of Graph pad software (Graph Pad Software, San Diego, CA, USA). Two groups were compared with an unpaired Student’s t test and two-tail p value. Results were considered statistically significant when p < 0.05.

## Results

### UVA irradiation leads to mitochondrial ROS-dependent autophagic cell death in RPE cells

**Fig. 1:**
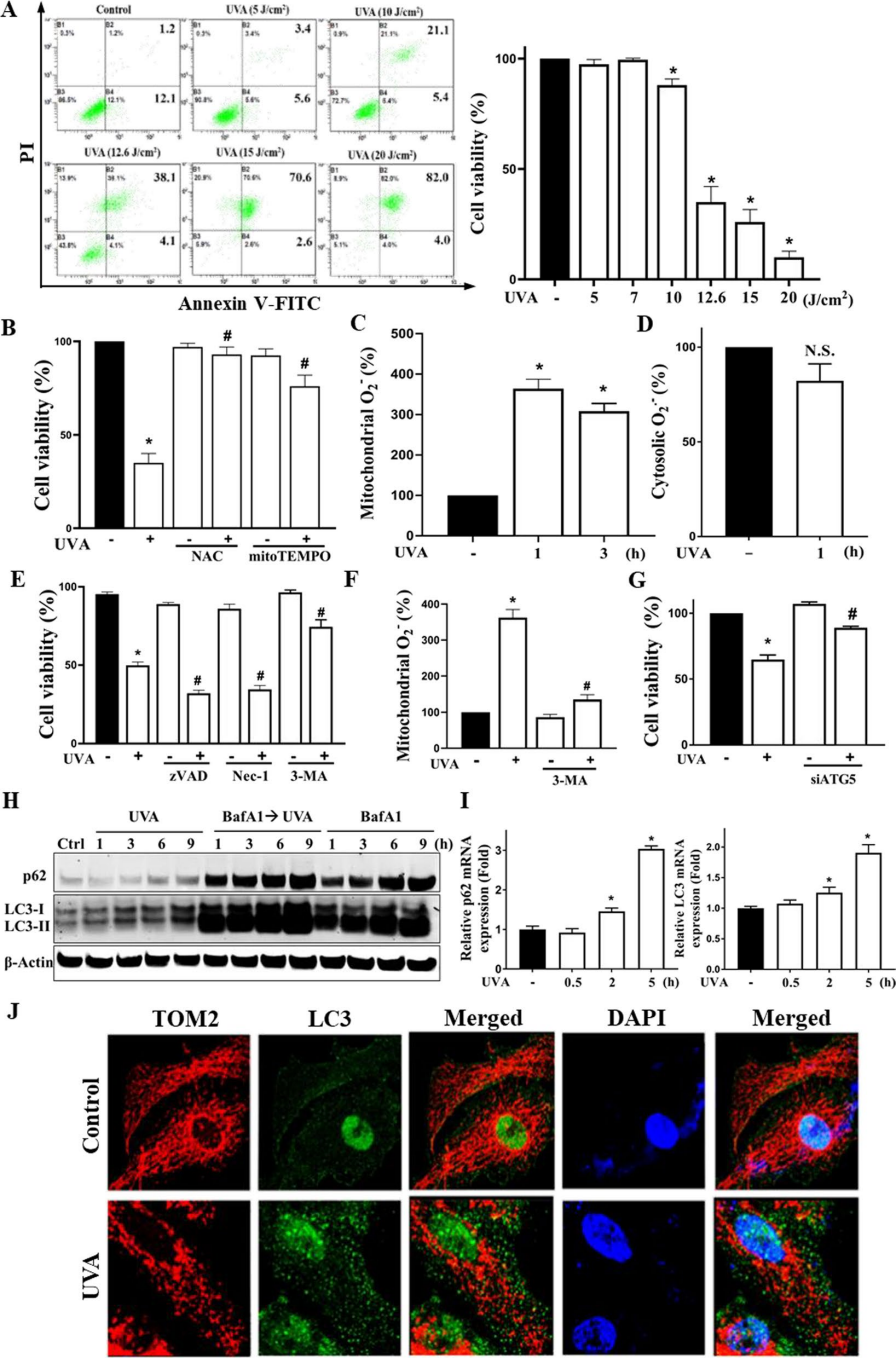
UVA-induced mitochondrial ROS production contributes to autophagic cell death in RPE cells. **A** Human ARPE-19 cells were subjected to UVA irradiation at different doses (5–20 J/cm^2^). **B–F** Cells were pretreated with vehicle, NAC (5 mM), mitoTEMPO (100 µM), zVAD (10 µM), necrostatin-1 (10 µM), or 3-MA (5 mM) 30 min prior to UVA (12.6 J/cm^2^) irradiation. **G** Cells were treated with ATG5 siRNA before UVA irradiation. Cell viability in **A, B, E, G** was determined by Annexin V-FITC/PI staining followed by flow cytometry analysis at 18 h post-UVA irradiation. **C, D, F** After 1 h **(C, D, F)** and/or 3 h **(C)** post-UVA irradiation, mitochondrial ROS (**C, F**) and cytosolic ROS (**D**) were determined using mitoSOX and DHE staining, respectively. **H** ARPE-19 cells were pretreated with bafilomycin A1 (100 nM) 60 min prior to UVA (12.6 J/cm^2^) irradiation. Immunoblotting of p62/SQSTM1 and LC3-I/-II expression was determined at the indicated times (1, 3, 6, and 9 h) post-UVA irradiation. **I** Real time PCR was used to determine p62/SQSTM1 and LC3 gene expression at 0.5, 2, and 5 h post-UVA irradiation. **J** Confocal laser microscopic images of TOM20, LC3 and DAPI in ARPE-19 cells at 1 h post-UVA (12.6 J/cm^2^) irradiation. Data were the mean ± S.E.M. of at least three independent experiments. *p < 0.05, indicating the significant effects of UVA; ^#^p < 0.05, indicating the significant effect of pretreatment to either reduce or enhance the effect of UVA; N.S., not significant

To establish the UV-damaged model of RPE cells, we first tested the phototoxicity with different intensities of UVA in ARPE-19 cells. We found that after exposure to UVA for 18 h, there existed an intensity-dependent (5–20 J/cm^2^) RPE cell death as indicated by the Annexin V-PI assay (Fig. 1A). We chose the 12.6 J/cm^2^, which maintained around 50% of viability post-UVA stimulation, as the final intensity for following phenotypic and mechanistic studies. UV illumination has been reported to generate ROS, and the high level of ROS accumulation leads to cell death. Therefore, we pretreated the cells with either the universal ROS scavenger NAC (5 mM) or the mitochondrial ROS-specific scavenger mitoTEMPO (100 µM) to investigate the role of ROS. As Fig. 1B showed, NAC exerted a complete protection and mitoTEMPO also induced a significant protection by about 80%. These data suggest that mitochondrial ROS are involved in the cell death pathway elicited by UVA. Further analysis with the ROS-specific dye MitoSOX revealed that the mitochondrial ROS level was significantly increased at 1 h after UVA (Fig. 1C), whereas the cytosolic ROS level exhibited no significant alteration at the same time point (Fig. 1D).

To understand the death mode of UVA, we treated cells with caspase inhibitor zVAD (10 µM), necroptosis inhibitor necrostatin-1 (10 µM), and autophagy inhibitor 3-methyladenine (3-MA, 5 mM). As shown in Fig. 1E, the cytotoxicity caused by UVA was not inhibited by zVAD or necrostatin-1, but was dramatically protected by 3-MA. Moreover, 3-MA was able to reduce UVA-induced mitochondrial ROS production (Fig. 1F). These data suggest that autophagy is involved in the ROS-dependent cell death pathway under UVA exposure. Confirming the role of autophagy, RPE cells were also protected by knockdown of ATG5 (Fig. 1G). In addition to the biochemical assay, we also ascertained the response of the whole autophagic flux by testing the mRNA and protein expression of p62 and LC3-I/-II. The p62 protein appeared to be accumulated at 6 h post-UVA exposure, while the LC3-II kept increasing within 9 h. After treatment with lysosome vATPase inhibitor, bafilomycin A1 (BafA1), both protein levels were markedly accumulated as expected. Meanwhile, under the treatment with BafA1, UVA-induced p62 and LC3 expression were further increased (Fig. 1H). These findings suggest the ability of UVA to induce autophagic flux. On the other hand, the p62 and LC3 mRNA levels started increasing at 2 h post UVA exposure followed by drastically elevating at 5 h post UVA exposure (Fig. 1I). Furthermore, the confocal laser scanning microscopy images of the immunocytochemistry in ARPE-19 cells co-staining with the mitochondrial marker (TOM20) and the autophagic marker (LC3) indicated that 1 h after UVA-illumination did cause the mitochondria fission as well as the increase of LC3 punctate, the typical marker of autophagy (Fig. 1J). Notably, no mitophagy was observed as there was no co-localized signal between TOM20 and LC3, which has been reported to be one of the mitophagy phenotypes. In summary, UVA-illumination increases the mitochondrial ROS production which eventually leads to the autophagic cell death in RPE cells.

### AMPK activators A769662 and metformin protect UVA-induced autophagic death via decreasing mitochondrial fission, ROS production and autophagy

**Fig. 2:**
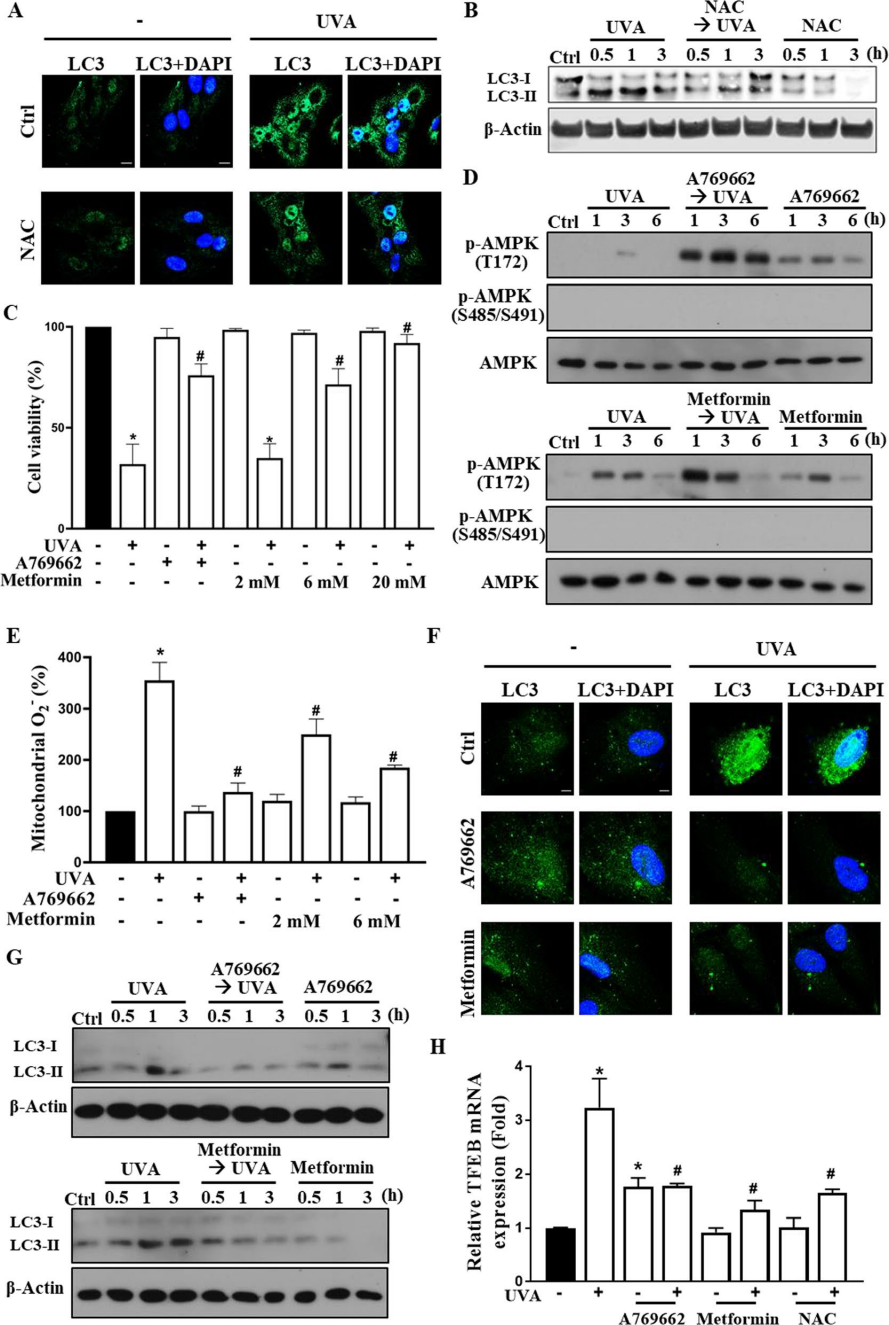
A769662 and metformin protect RPE cells from UVA-induced cell death. ARPE-19 cells were pretreated with NAC (5 mM) (**A**, **B**, **H**), A769662 (25 µM) or metformin (6 mM unless otherwise indicated) (**C**–**H**) 30 min prior to UVA (12.6 J/cm^2^) irradiation. **A, F** Confocal microscopic images of LC3 and DAPI in cells 1 h post-UVA irradiation. **B, D, G** At 0.5, 1, 3 and 6 h post-UVA irradiation, cell lysates were harvested for immunoblotting. **C** Cell viability was determined at 18 h post-UVA irradiation by Annexin V-FITC/PI staining. **E** Mitochondrial ROS level was detected by MitoSOX staining at 1 h post-UVA irradiation. **H** Real time PCR was used to determine TFEB gene expression at 5 h post-UVA irradiation. Data were the mean ± S.E.M. of at least 3 independent experiments. *p < 0.05, indicating the significant effect of UVA; ^#^p < 0.05, indicating the significant effects of pretreatment agents on the effects of UVA

After observing that UVA-induced autophagy contributes to ROS-dependent cell death, we further examined the association between ROS and autophagy induction. As shown in Fig. 2A and B, the data from immunocytochemistry and western blot revealed that the UVA-induced LC3 punctate and LC3-II protein expression were reduced by NAC (5 mM), suggesting that ROS-autophagy axis mediates UVA irradiation-induced RPE cell death. Next, we would like to elucidate the role of AMPK, an upstream molecular signal of autophagy, in the UVA-induced death event. We first applied the AMPK activators A769662 and metformin to the ARPE-19 cells prior to UVA-illumination. Surprisingly, A769662 (25 µM), as well as metformin above 6 mM, exhibited a protection effect against the UVA-induced cell damage instead of enhancing the cell death by AMPK activation (Fig. 2C). Further investigation of AMPK status, we demonstrated that UVA can moderately increase AMPK phosphorylation at T172 and this effect was markedly enhanced under metformin or A769662 treatment (Fig. 2D). On the other hand, the inhibitory AMPK phosphorylation at S485/S491 was not observed under these treatments (Fig. 2D). These data indicate that AMPK is activated by UVA, A769662, and metformin.

To further solve the role of AMPK in cell death, we measured the effects of A769662 and metformin on mitochondrial ROS and autophagy as mitochondrial ROS-mediated autophagic cell death is proposed above. We found that both agents attenuated UVA-induced mitochondrial ROS production (Fig. 2E). Moreover, we observed that the A769662 and metformin pretreatments reduced the autophagy activity post-UVA exposure as indicated by the immunocytochemistry of LC3 staining (Fig. 2F) and immunoblotting probed with anti-LC3 antibody (Fig. 2G). In Fig. 2H, the qRT-PCR of the transcription factor EB (TFEB), a well-known regulatory factor in autophagosome and lysosome-related genes activation, also indicated that TFEB gene expression that upregulated by UVA after 5 h was reduced in A769662, metformin, and NAC pretreatment groups. These findings suggest that activation of AMPK prior to UVA exposure can protect cells *via* suppressing mitochondrial ROS production and autophagy.

### AMPK activators reverse the mitochondrial fragmentation and membrane potential loss without changing oxidative phosphorylation in UVA-damaged RPE cells

**Fig. 3:**
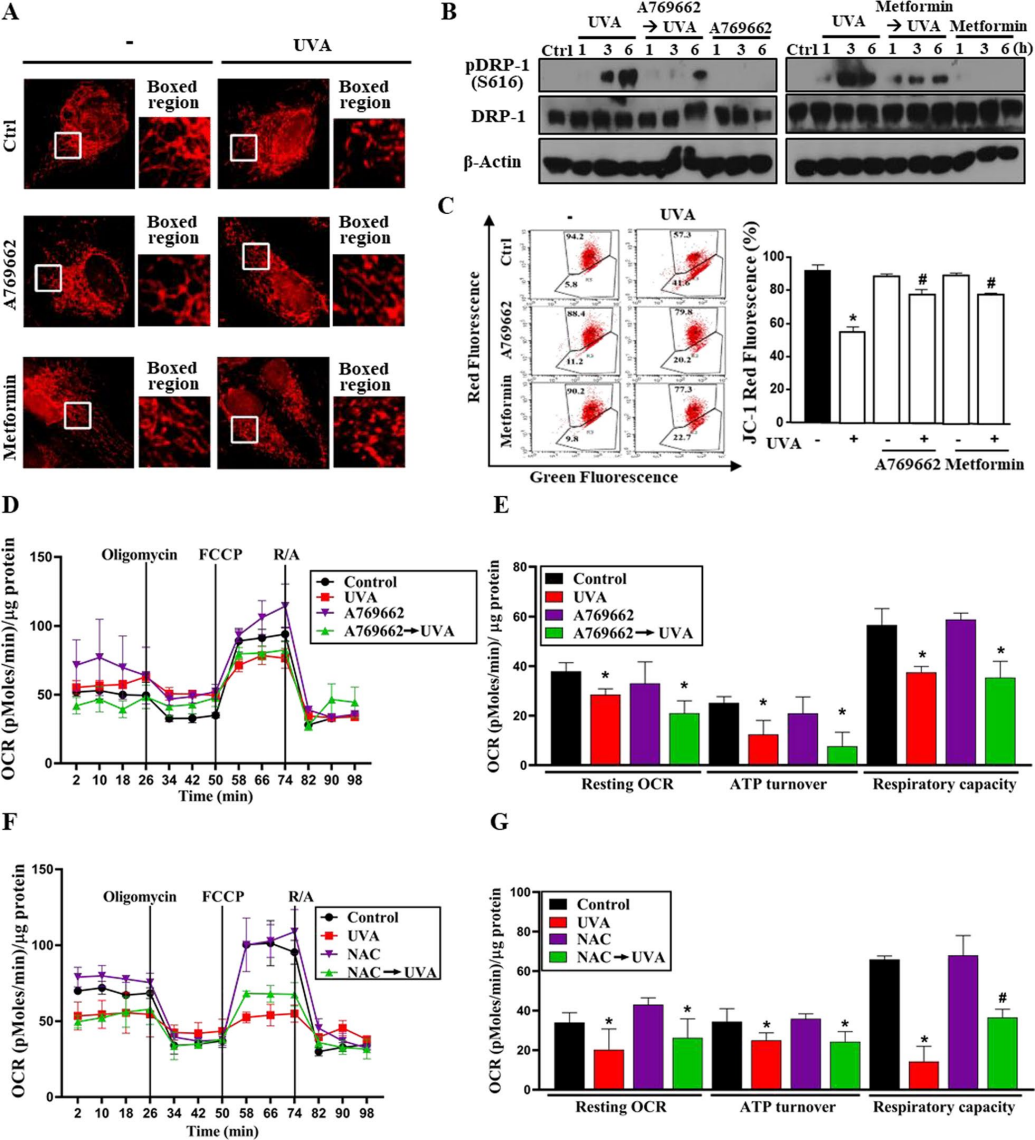
AMPK activators inhibit UVA-induced mitochondria fragmentation and MMP loss without affecting the inhibition on mitochondrial oxidative phosphorylation. ARPE-19 cells were pretreated with AMPK activators A769662 (25 µM), metformin (6 mM), or NAC (5 mM) 30 min prior to UVA (12.6 J/cm^2^) irradiation. **A** Confocal microscopic images were performed at 1 h post-UVA irradiation. Mitotracker-Red CMXRos was used to detect the morphology of the mitochondria. **B** At the indicated time points after UVA irradiation cells were harvested by sample loading buffer followed by immunoblotting. **C** Mitochondrial membrane potential was determined by JC-1 staining at 1 h-post UVA irradiation. **D–G** Seahorse assay was performed for measuring mitochondrial OXPHOS in RPE cells after 1 h treatment with UVA. Data were the mean ± S.E.M. of 3 independent experiments. *p < 0.05, indicating the significant effects of UVA. ^#^p < 0.05, indicating the significant effects of A769662 and metformin to reverse UVA actions

Knowing that the AMPK activators exert a protective effect in UVA-damage RPE cells by inhibiting ROS-dependent autophagy, we further dissected their effects on mitochondria in addition to mitochondrial ROS level. Examination of mitochondrial morphology by the immunocytochemistry staining with MitoTracker revealed the abilities of A769662 and metformin pretreatment to reverse the mitochondria fission at 1 h post-UVA exposure (Fig. 3A). A similar trend of reversed mitochondrial fission was also observed from immunoblotting on DRP1 phosphorylation at S616, a mitochondrial fission marker (Fig. 3B). Moreover, a rescued effect on mitochondrial membrane potential (MMP) was observed in A769662 and metformin pretreating groups (Fig. 3C). Seahorse assay which shows the mitochondrial oxidative phosphorylation status was performed to examine the metabolic function post-UVA exposure. Notably, UVA can rapidly reduce oxygen consumption rate after 1 h stimulation and the reduction of resting OCR, ATP turnover and respiratory capacity cannot be affected by pretreating A769662 (Fig. 3D, E) or NAC (Fig. 3F, G), suggesting that the UVA-damaged metabolic function is not associated with mitochondrial dynamics controlled by ROS-AMPK. Taken together, AMPK pre-activation exerts a protection effect against the UVA-induced RPE cell death by maintaining the mitochondrial dynamics and mitochondrial membrane potential but attenuating the mitochondrial ROS production.

### Mitochondrial ROS-dependent PARP-1 activation reduces UVA-induced DNA damage and cell death

**Fig. 4:**
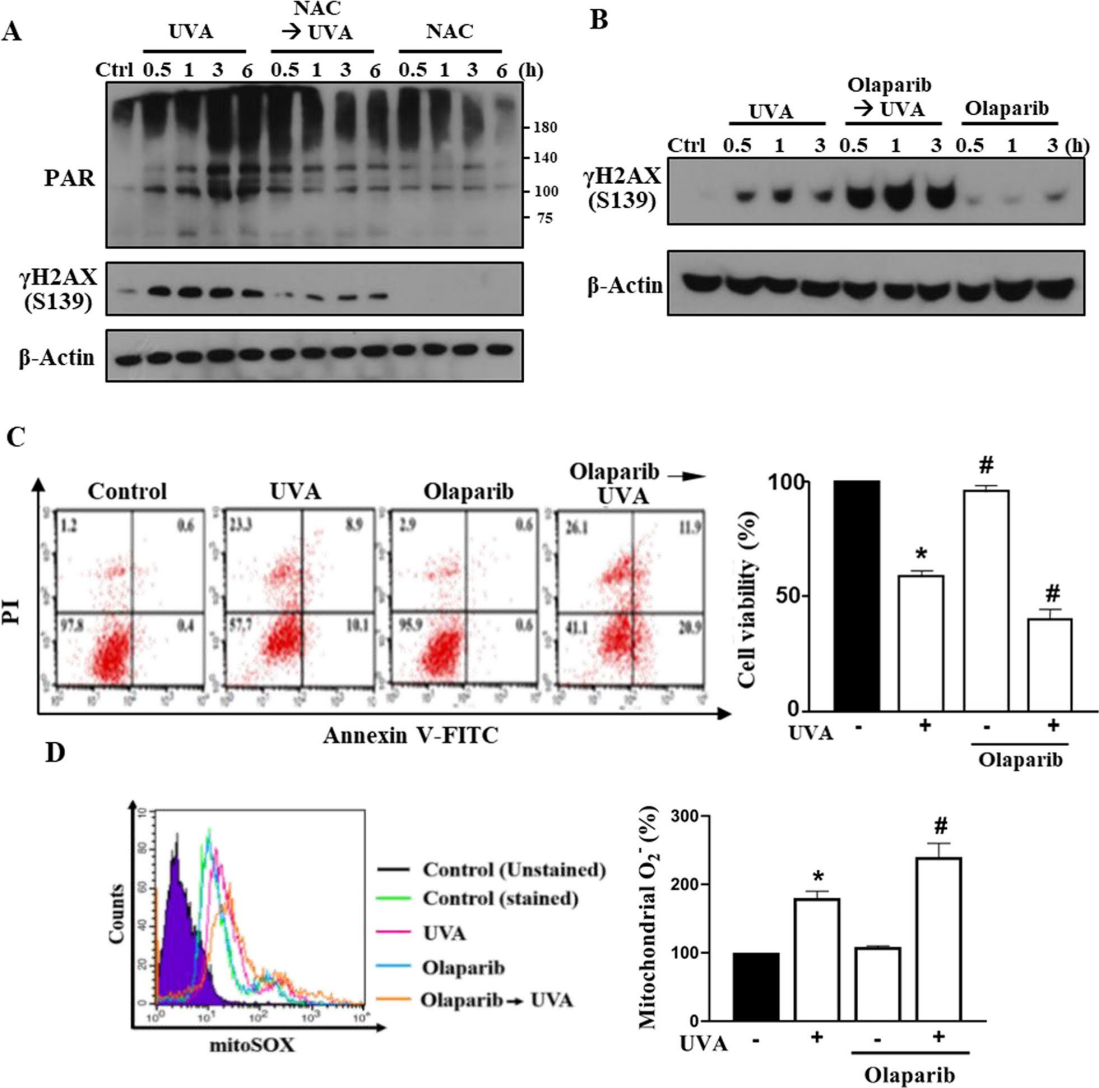
UVA-induced PARP-1 activation can reduce DNA damage and subsequent cell death. ARPE-19 cells were pre-treated with NAC (5 mM) (**A**) or olaparib (10 µM) (**B**–**D)** 30 min prior to UVA (12.6 J/cm^2^) irradiation. **A, B** After UVA exposure at the indicated time points cell lysates were collected for immunoblotting. **C** Cell viability was determined by Annexin V-FITC/PI at 18 h post-UVA irradiation. **D** Mitochondrial ROS level was detected by MitoSOX staining at 1 h after UVA. Data were the mean ± S.E.M. of 3 independent experiments. *p < 0.05, indicating the significant effects of UVA; ^#^p < 0.05, indicating the significant effects of olaparib to enhance cell death and increase ROS production

Because UV irradiation induces DNA damage-associated death events, we further ascertained the DNA damage status in RPE cells post-UV illumination. We found that NAC pretreatment can attenuate the expression of DNA repair marker (i.e. PARylation mediated by PARP-1 activation) and the double-strand break DNA damage marker (γH2AX) post-UVA illumination (Fig. 4A). In contrast, pretreatment of the PARP inhibitor olaparib (10 µM) resulted in a much higher expression of γH2AX (Fig. 4B) and an enhanced cellular death (Fig. 4C) post-UVA illumination. Further investigation of the mitochondrial ROS level demonstrated a higher mitochondrial ROS production in RPE cells pretreated with olaparib (Fig. 4D). These findings indicate a consequential link between mitochondrial ROS and DNA damage, which in turn leads to PARP-1 activation for DNA repair.

### AMPK activators inhibit UVA-induced DNA damage and AMPK nuclear export

**Fig. 5:**
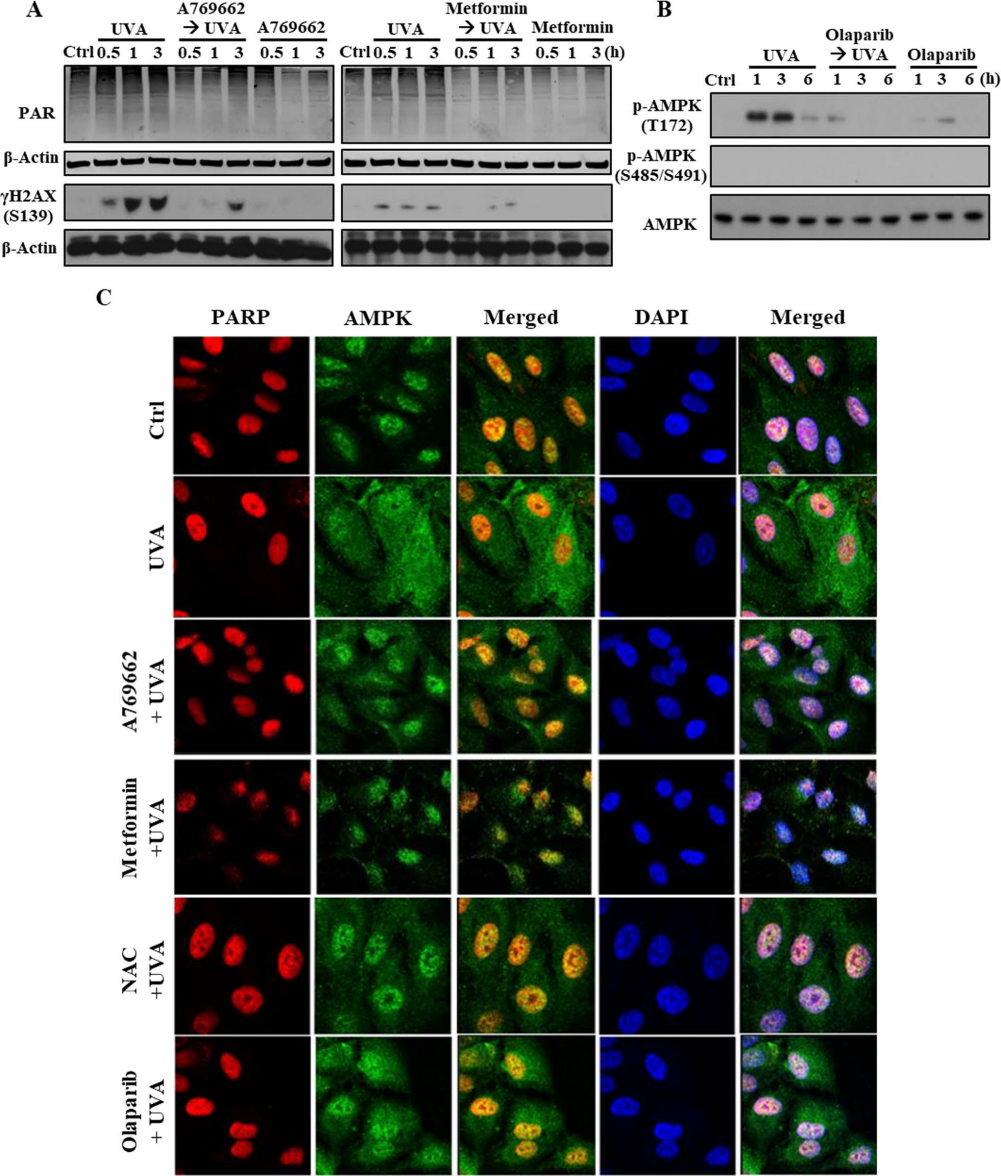
A769662 and metformin inhibit UVA-induced DNA damage and AMPK nuclear export. **A, B** ARPE-19 cells were pre-treated with AMPK activators A769662 (25 µM),  metformin (6 mM) (**A**) or olaparib (10 µM) **(B)** 30 min prior to UVA (12.6 J/cm^2^) irradiation. Cells lysates were collected at the indicated time points post-UVA irradiation for immunoblotting analysis. **C** ARPE-19 cells were pretreated with A769662 (25 µM), metformin (6 mM), NAC (5 mM), or olaparib (10 µM) 30 min prior to UVA irradiation. Cells were fixed at 1 h post-UVA irradiation for confocal microscopic analysis of PARP-1 and AMPK. Data were the representative of 3 independent experiments

Given that A769662 and metformin can protect RPE cells from UVA-induced mitochondrial ROS production, MMP loss, mitochondrial fission, and autophagy, and that PARP-1 is involved in DNA repair for minimizing cell death response, we then looked into the link between AMPK and PARP-1. Immunoblotting results indicated that both A769662 and metformin pretreatment reduced the UVA-induced DNA damage and PARylation (Fig. 5A), suggesting that the ROS-PARP-1 axis upon UVA exposure is blocked by AMPK activation. In addition, olaparib can inhibit UVA-induced AMPK activation as indexed of AMPK phosphorylation at T172 (Fig. 5B), further suggesting that UVA-induced AMPK activation depends on PARP-1. Another interesting issue we like to check is the subcellular localization of AMPK, as it is also an initiator of autophagy beyond the action in ROS reduction. The immunocytochemistry images with co-staining PARP-1 and AMPK unexpectedly showed that both molecules were major co-localized in the nuclei and UVA stimulation can rapidly trigger AMPK translocation from the nuclei to the cytosol within 1 h. Moreover, NAC, A769662, metformin, and olaparib pre-treatment restricted the AMPK nuclear export under UVA exposure (Fig. 5C). These findings indicate the ROS-PARP-1-AMPK activation pathway contributes to AMPK translocation from the nuclei to the cytosol for autophagy induction.

### Cathepsin B and lysosome dysfunction following autophagic stress contribute to cell death independent of ROS-AMPK axis

**Fig. 6:**
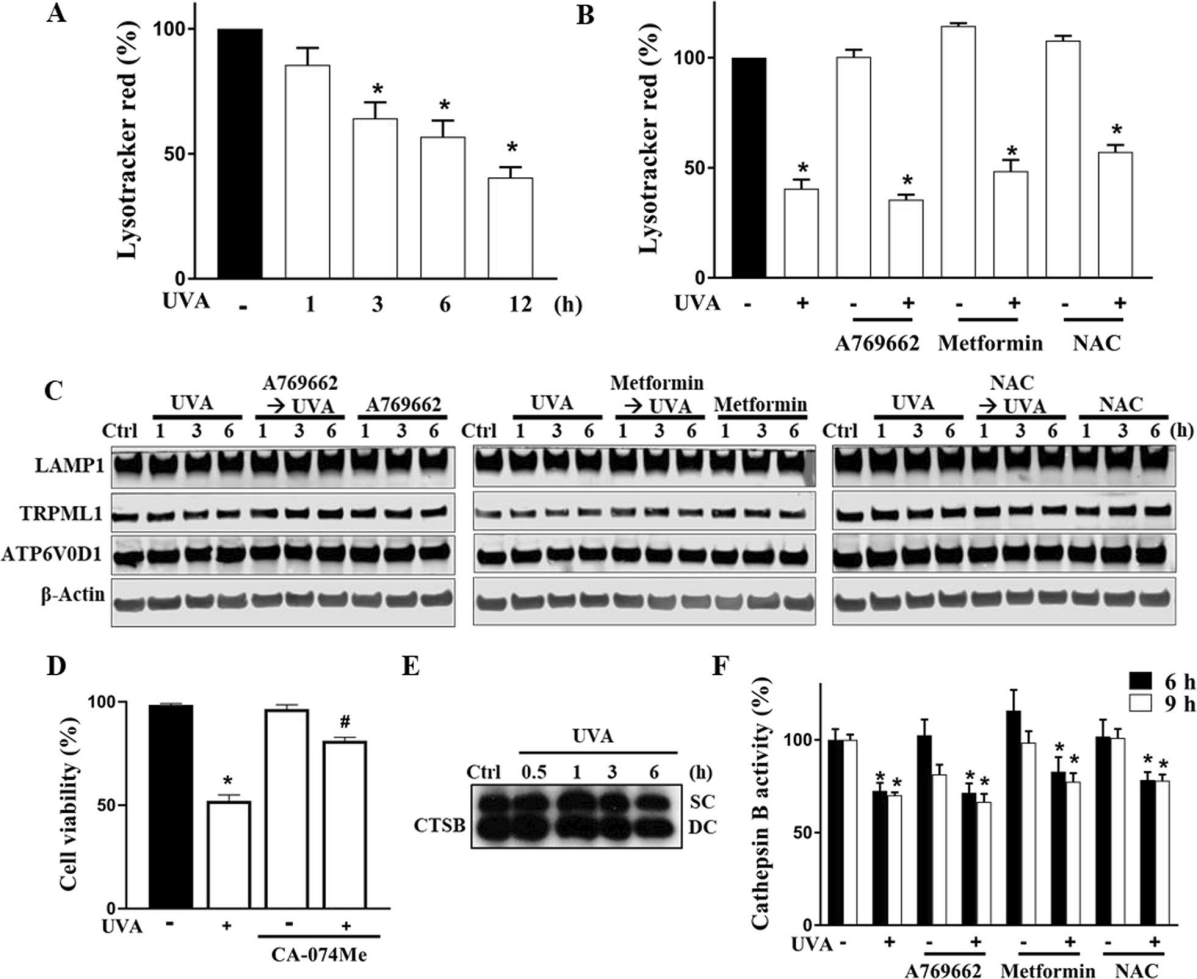
Cathepsin B is involved in cell death and UVA-induced gradual lysosome dysfunction is independent of ROS-AMPK axis. **A** At the indicated times (1, 3, 6, and 12 h) post-UVA irradiation lysosomes in ARPE cells were determined by flow cytometry with LysoTracker. **B–D, F** ARPE-19 cells were pretreated with A769662 (25 µM), metformin (6 mM), NAC (5 mM) or CA-074Me (10 µM) 30 min prior to UVA (12.6 J/cm^2^) irradiation. In **B** cells were harvested at 12 h post-UVA irradiation for flow cytometry analysis with LysoTracker. In **C** at 1, 3, and 6 h after UVA, immunoblotting was conducted. In **D** cell viability was determined by Annexin V-FITC/PI at 18 h after UVA. In **F** cathepsin B activity was determined using flow cytometry analysis with MagicRed at 6 and 9 h after UVA. **E** Immunoblotting of cathepsin B expression was determined at the indicated times (0.5, 1, 3, and 6 h) post-UVA irradiation. Data were the mean ± S.E.M. of 3 independent experiments. *p < 0.05, indicating the significant effects of UVA. ^#^p < 0.05, indicating the significant effect of CA-074Me to protect UVA-induced cell death

Autophagy comprises autophagic flux for autophagosome formation followed by lysosome fusion to accomplish the autophagic degradation. As we have demonstrated that ROS-AMPK-autophagy contributes to UVA-induced cell death and exogenous pre-activation of AMPK can attenuate cell death *via* the reduction of ROS level and AMPK translocation to cytosol, the role of the lysosome remains unexplored. We then started with LysoTracker staining to test the lysosomal mass. We found that at 1, 3, 6, and 12 h post-UVA irradiation, the overall lysosome mass detected by LysoTracker was decreased in a time-dependent manner (Fig. 6A). Surprisingly, when we pretreated ARPE-19 cells with A769662, metformin, or NAC, they did not reverse the decreased lysosomal mass at 12 h post-UVA irradiation (Fig. 6B). Because LysoTracker fluorescence is relying on the acidity and mass of lysosomes, and the latter might be resulting from the lysosomal biogenesis and lysosomal rupture after autophagolysosome formation, we further determined several lysosome marker proteins to differentiate the status on lysosomal mass and acidity. Immunoblotting analyses of lysosome-associated membrane protein 1 (LAMP1), transient receptor potential cation channel, mucolipin subfamily, member 1 (TRPML1), and vacuolar ATPase H^+^ transporting V0 subunit d1 (ATP6V0D1) revealed no significant changes of these protein expressions after UVA, A769662, metformin and/or NAC treatments (Fig. 6C). In addition, we measured lysosomal hydrolase cathepsin B expression and activity and determined its role in UVA-induced cell death. With the cathepsin B inhibitor CA-074Me (10 µM) pretreatment, we observed a protection effect toward UVA-induced cell death (Fig. 6D). We found UVA did not alter the total protein expression of single or double chain of cathepsin B (Fig. 6E). Moreover, the data of cathepsin B activity assay at 6 and 9 h post-UVA irradiation indicated a loss of the enzymatic activity, while such inhibition was not altered by the treatment with A769662 (25 µM), metformin (6 mM) or NAC (5 mM) (Fig. 6F). Meanwhile, at early time points before 6 h, we did not observe a significant change on cathepsin B activity under UVA stimulation (data not shown). Taken together, cathepsin B activity contributes to UVA-induced cell death, and UVA-induced late lysosomal dysfunction is independent of autophagic flux that occurs rapidly *via* the ROS-PARP-1-AMPK pathway.

### EGFR inhibition enhances UVA-induced cell death via increasing autophagy and lysosomal dysfunction

**Fig. 7:**
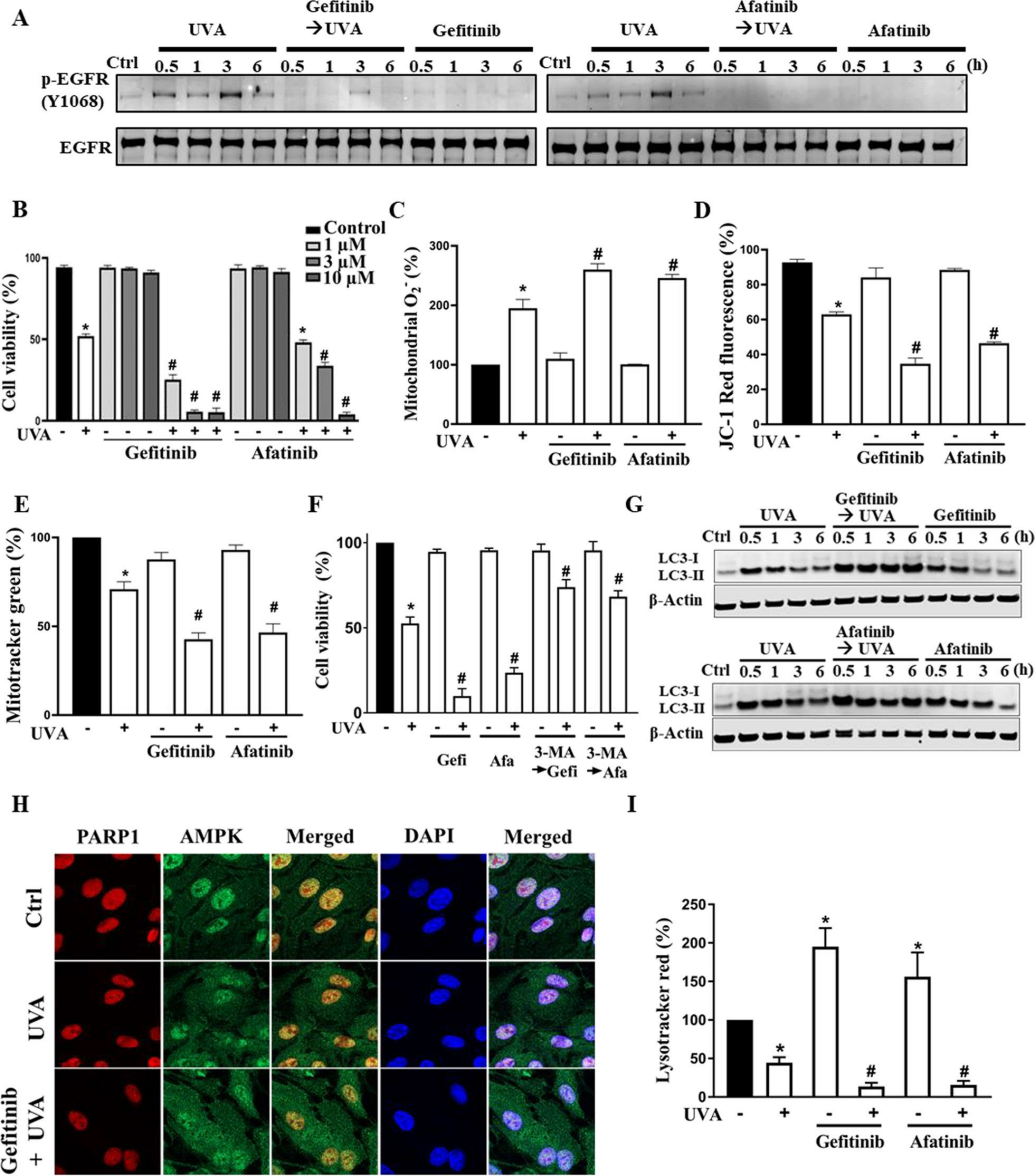
UVA-induced EGFR transactivation reduces cell death by exerting dual actions in balancing autophagic flux and lysosomal dysfunction. Cells were pretreated with gefitinib (gefi; 1 µM), afatinib (afa; 3 µM), or 3-MA (5 mM) 30 min prior to UVA (12.6 J/cm^2^) irradiation. **A, G** At 0.5, 1, 3, and 6 h after UVA immunoblotting was performed. **B, F** Cell viability was determined by Annexin V-FITC/PI staining at 18 h post-UVA irradiation. **C, D** Mitochondrial ROS level and MMP were determined at 1 h after UVA by flow cytometry with MitoSOX and JC-1 staining, respectively. **E, I** Mitochondrial mass and lysosomal mass were determined at 6 h after UVA by flow cytometry with MitoTracker and LysoTracker staining, respectively. Data were the mean ± S.E.M. of 3 independent experiments. *p < 0.05, indicating the significant effects of UVA; ^#^p < 0.05, indicating the significant effects of drug pretreatments on UVA-induced responses as compared to vehicle-treated cells. **H** ARPE-19 cells were pretreated with gefitinib (1 µM) 30 min prior to UVA irradiation. Cells were fixed at 1 h post-UVA irradiation for confocal microscopic analysis of PARP-1 and AMPK. Data were the representative of 3 independent experiments

Autophagy is negatively controlled by the AKT/mTOR signaling pathway, and EGFR has been shown to be activated by UV in keratinocytes and skin [35, 37, 38] and regulate autophagy via PI3K/AKT/mTOR in different cell types including keratinocytes [39]. Next, we were interested in determining if UVA irradiation can affect EGFR activity in ARPE-19 cells. As shown in Fig. 7A, UVA can induce EGFR transactivation as indexed by the increased EGFR phosphorylation at Y1068 and the block of this effect by two EGFR tyrosine kinase inhibitors (TKIs) gefitinib (1 µM) and afatinib (3 µM). With gefitinib or afatinib treatment, we observed a concentration-dependent enhancement of cytotoxicity post-UVA irradiation at a concentration range of 1–10 µM (Fig. 7B). The enhanced cell death post-UVA irradiation was also accompanied by increased mitochondrial ROS production (Fig. 7C), reduced MMP (Fig. 7D) and reduced mitochondrial mass (Fig. 7E). To further dissect the roles of autophagosome and lysosome under the condition of EGFR-TKIs pretreatment, we tested the 3-MA and EGFR-TKIs combination. We found that the protection effect of 3-MA in UVA-induced cell death was also detected in the TKIs-treated cells (Fig. 7F), confirming our previous notion of UVA-induced autophagic death. Furthermore, the immunoblotting demonstrated a further elevated level of LC3-II in TKIs-treated cells (Fig. 7G). In addition to elevated LC3-II expression, immunocytochemistry images of gefitinib (1 µM) pretreatment exhibited a more cytosolic than nuclear AMPK distribution at 1 h post-UVA illumination (Fig. 7H). On the other hand, the flow cytometry data showed that at 6 h incubation, EGFR-TKIs themselves can increase LysoTracker signal, but further reduce the effect of UVA (Fig. 7I). Altogether, UVA-induced EGFR transactivation exerts dual actions to compromise ROS-AMPK-autophagy axis and lysosomal dysfunction induced by UVA irradiation. Removing this counteracting EGFR signal pathway leads to a deterioration in autophagosome accumulation and lysosome dysfunction, and then an enhanced cell death.

## Discussion

UV radiation exposure from sunlight is the major risk factor for the development of skin cancer, skin photoaging, and retinopathy. As an oxidizing agent, UVA causes significant damage to cellular components through the production of ROS, leading to photoaging. To date, UV-induced cell damage, photoaging, and even photocarcinogenesis are well investigated in skin, while only a few studies are demonstrated in retinal, especially RPE cells. In this study, we found mitochondrial ROS production mediates cell death in RPE cells after UVA exposure. Although a previous study by Yao et al. showed that UV can induce AMPK-dependent ARPE-19 cell apoptosis, what they used was UV at 25 mJ/cm^2^ rather than UVA [40]. Likewise, UVB and UVC have been reported to induce apoptosis in ARPE-19 cells [41, 42]. Therefore, different wavelengths of UV induce variable cellular events and death modes in RPE cells. Meanwhile, we for the first time observed several new findings in RPE cells. First, UVA induces autophagy-associated cell death in human RPE cells via ROS-PARP-AMPK-autophagic flux and cathepsin B activation. Second, pretreatment of AMPK activators, a positive regulator of autophagy, exerts a protection in UVA-damaged RPE cells by reducing the mitochondrial ROS. Third, lysosome dysfunction at the mid to late phase after UVA insult contributes to cell death and is independent of AMPK. Fourth, UVA transactivates EGFR which further balances autophagy and lysosomal dysfunction in the UV-stressed condition.

Autophagy is an evolutionary cellular homeostatic process to catabolically clear unwanted or damaged proteins, lipids, and organelles [5, 43]. In RPE cells, autophagy plays a crucial role in the daily-based clearance of photoreceptor outer segments to maintain the homeostasis of RPE [44]. Knockout of the *Atg5* and *Atg7* in mice RPE results in insufficient autophagy and age-related macular degeneration-like phenotype in aged mice [45]. However, autophagy is a double-edged sword that possesses both pro-survival and pro-cell death effects. UV-induced autophagy has been reported to inhibit the death of skin cells [19–21]. In our study, we observed an increased induction of AMPK phosphorylation at 0.5–3 h post UVA exposure as well as the elevation of autophagy marker (LC3-II), suggesting an initiation of autophagic flux. However, at the mid-to-late phase (> 6 h post-UVA exposure), LC3-II and p62 are upregulated time-dependently, implying the insufficiency or potential impairment of the lysosomes ends up leading to an autophagolysosome accumulation and autophagic cell death.

UV exposure triggers a cascade of events that lead to DNA damage, cell death, or DNA repair. Moreover, DNA damage reciprocally linked to ROS increase is the underlying mechanism for cell death caused by UVA [1]. In this study, we confirmed this scenario on the tight link between ROS and DNA damage. We found mitochondrial ROS, but not cytosolic ROS, production plays the key in UVA-induced RPE cell death. Attenuation of mitochondrial ROS by non-specific antioxidant NAC and mitochondria-specific antioxidant mitoTEMPO confers cell protection. Furthermore, we showed increased mitochondrial ROS level and DNA damage in UVA-stressed RPE cells co-treated with olaparib. Taken together, our study strengthens the tight crosstalk between ROS and PARP-1 in RPE cells in response to DNA damage under UVA stress. Currently, we still do not understand the reason for no cytosolic ROS production after UVA irradiation. Nevertheless, previously we also observed increased mitochondrial ROS but not cytosolic ROS production in other stress conditions in RPE cells (e.g. methylglyoxal) [46]. DHE is the most commonly used reagent to present cellular (cytosolic) ROS, especially for O_2_^−^, while it might not be able to detect different types of ROS. This might be one of the reasons for the inconsistency in the compartmental ROS measurements.

UV exposure has been demonstrated to trigger AMPK-dependent cell protective autophagy in skin cells [19–22]. Likewise, in UVA-stressed RPE cells, we also observed a rapid activation of AMPK which is mediated by mitochondrial ROS. One of the most striking observations in this work is the ROS-PARP-1 axis-mediated AMPK nuclei-to-cytosol translocation. Since AMPK-mediated autophagic flux majorly occurs in the cytosol, we suggest that AMPK nuclear export is essential to initiate autophagic flux. This PARP-1-dependent AMPK nuclear export for autophagy induction is not widely reported, to date has only been reported in breast cancer cells and fibroblasts upon starvation [47]. To date, several studies demonstrate that autophagy can help DNA damage repair upon UV radiation [18, 24]. Among them, one is that autophagy can positively regulate the recognition of DNA damage by increasing XPC transcription and DDB2 recruitment to the CPD site [23]. AMPK is a multifunctional protein kinase, and in addition to autophagy induction it is involved in promoting DNA repair upon DNA damage [48] and subsequent skin tumorigenesis [24, 28].

AMPK has also been reported to be involved in redox regulation, mitochondrial dynamics, and mitochondrial ROS homeostasis. The underlying mechanisms for these events include promoting the AMPK/Nrf2/Sirt3 pathway, inhibiting the ERK-Akt signal axis dependent Drp-1 phosphorylation at S616 of mitochondrial fission pathway, and protecting the mitochondria electron transfer chain, respectively [49–51]. A769662 and metformin have been widely used to activate AMPK and metformin even has been reported as a potential treatment for retinal disease such as diabetic retinopathy [52]. Moreover, our previous study demonstrated that A769662 can protect the RPE cells against NaIO_3_ [50]. As mentioned above, even though UVA-induced AMPK activation leads to autophagic cell death, our present findings indicate a new intervention to mitigate UVA stress by activating AMPK before insult. When manipulating AMPK activity by pretreating AMPK activators, a significant cell protection is observed. Mechanistic investigation indicates such protection mainly results from the attenuation of ROS production and homeostatic regulation of mitochondrial dynamics.

In addition to the autophagosome forming axis, the other critical part of autophagy is the autophagosome and lysosome fusion to accomplish autophagic flux. Autophagosome forms and captures the damaged organelles and/or malfunctioned biomaterials (e.g., nucleotides, proteins, and lipids) and then fuses with lysosome for degradation of the cargoes inside autophagosome via hydrolases (e.g., cathepsin B/D) to maintain the cell homeostasis [53]. Besides mitochondrial defects, lysosomal dysfunction can be induced by mitochondrial ROS and lead to autophagy impairment [54]. In response to UV irradiation, both mitochondria and lysosomes are easily damaged resulting from oxidative stress [55]. Cathepsin B is a cysteine protease and is primarily localized within the lysosomal compartment. Interests are growing in cathepsin B due to its diverse roles in physiological and pathological processes, especially in mediating various modes of programmed cell death [56]. Recent studies indicate that cathepsin B provides a checkpoint for homeostatic maintenance of lysosome populations. Nevertheless, during lysosomal stress in autophagic cell death condition, autophagic-lysosomal dysregulation would reduce cathepsin B activity, induce lysosomal leakage, and increase autophagosome accumulation [56, 57]. In turn, cathepsin B inhibition can drive autophagy via at least two mechanisms. First, cathepsin B inhibition upregulates TFEB gene transcription via stabilization of lysosomal calcium channel TRPML1, leading to an increase in the expression of lysosomal and autophagy-related proteins. Second, the activity of mTOR, which is an autophagy inhibitory signaling pathway, is positively controlled by cathepsin B [58, 59]. Therefore, our finding that UVA irradiation-induced reduction of cathepsin B activity in RPE cells agrees with previous study in UV-stimulated skin fibroblasts [57]. We also found such reduced enzymatic activity of cathepsin B is not due to altered protein expression. In this study, we found this stress-induced cathepsin B inhibition occurring at 6 h after UVA irradiation is independent of ROS production or AMPK activation. Paradoxically, using cathepsin B inhibitor we observe the contribution of cathepsin B activity in autophagic death. In our previous study, we also observed the cathepsin B leakage at the late phase of P2X7-mediated microglial cell death, and this effect is AMPK-independent [36]. All these findings prompt us to suggest the existence of a cathepsin B-mediated death pathway beyond ROS-PARP-1-AMPK-autophagy. Pre-activation of AMPK can block the latter but not the former death pathway. In summary, we show that even though cathepsin B activity is inhibited by UVA independent of the ROS-autophagy axis, it is involved in autophagy-dependent cell death. It remains to be addressed how UVA decreases cathepsin B activity in parallel with the enhanced autophagic flux and how cathepsin B is involved in autophagy-associated cell death. In addition, if lysosomal membrane proteins are directly impaired, or if lysosomal stress results from ER stress are still needed for further investigation. But at least at this stage, the expressions of three lysosomal proteins including LAMP1, TRPML1, and ATP6V0D1 are not affected.

Another novel finding of this study is highlighting the role of EGFR in RPE cells. EGFR is a tyrosine kinase receptor located at the cell membrane. EGFR activation by UVB-stimulated skin or keratinocytes has been shown to induce inflammation [60, 61]. Even though EGFR transactivation can be induced by UV irradiation in keratinocytes [35], how EGFR is involved in UV-induced cell death is largely unknown. Some evidence also indicates that the engagement of the EGFR can induce proliferation, differentiation, and migration of RPE cells, contributing to proliferative vitreoretinopathy and blindness [31–34]. Previous studies indicate an autocrine/paracrine role for EGF, TGF-α, HB-EGF, and EGFR in proliferative diabetic retinopathy. Although EGFR activation by EGF treatment can protect ARPE-19 cells from H_2_O_2_-induced cell death [62, 63], we do not get this effect in UVA-stressed RPE cells (data not shown). Nevertheless, we indeed observed the ability of UVA to activate EGFR in RPE cells and confirmed the protective role of autocrine EGFR activation against UVA insult.

EGFR can modulate autophagy in different models, such as non-small cell lung cancer, brain tumors, and keratinocytes. This modulation is mainly mediated by the activation of the PI3K/AKT/mTOR pathway [39, 64, 65]. To have a more detailed analysis of the contribution of transactivated EGFR in UVA-induced autophagic cell death, we further determined the effects of EGFR TKIs gefitinib and afatinib. The findings reveal a more autophagosome formation with the lower lysosome mass/activity, leading to an increased RPE cell death post-UVA irradiation. This phenotype of the formation of autophagosomes during lysosomal defect has been reported to confer cytotoxicity in several cell types including the kidney, liver, retina, muscle, endocrine glands, and neurons [66]. The effects of both TKIs on enhancing cell death accompanied by the increased mitochondrial ROS production, mitochondria membrane potential loss, mitochondrial mass reduction,  and LC3 upregulation support our suggestion of a ROS-dependent autophagy pathway. However, different from AMPK activator pretreatment, TKIs deteriorate lysosome function in UVA stressed conditions. This finding agrees with previous observation of EGFR TKIs in regulating lysosome function [67]. TKIs treatment alone to increase lysosomal mass might result from the inhibition of mTOR which exerts a negative inhibition on TFEB-dependent lysosomal biogenesis [68]. The other possibility may come from the EGFR-TKI-induced autophagic flux activation, hence the increased lysosome mass [69].

## Conclusion

**Fig. 8:**
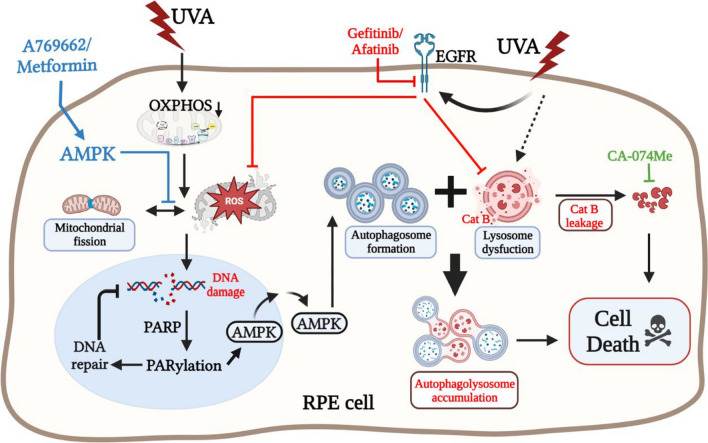
Spatiotemporal role of AMPK in regulating UVA-induced autophagy cell death in RPE cells. UVA-irradiation rapidly increases mitochondrial ROS production and DNA damage, leading to AMPK nuclear export and overactivated autophagic flux. At the mid to late phase, UVA also induces lysosome dysfunction (i.e. lysosomal rupture and leakage of cathepsin B), causing incomplete autophagy and autophagolysosome accumulation. Pre-activating AMPK by AMPK activators, on the other hand, protects RPE cells from UVA stress by reducing mitochondrial ROS production and the following signal cascades. EGFR transactivation by UVA also exerts balanced effects on autophagic flux and lysosome dysfunction. As such, EGFR TKI can deteriorate the UVA-induced cell death

We for the first time show the molecular mechanisms of UVA-induced autophagic death in RPE cells via ROS-PARP-1-AMPK and lysosomal cathepsin B pathways (Fig. 8). The rapid induction of photo-oxidative stress by UVA would target mitochondria and lead to mitochondrial damage and ROS production. In turn, ROS-dependent PARP-1 activation causes DNA repair and AMPK activation. AMPK localization majorly in the nuclei of RPE cells is activated by PARP-1, and its subsequent nuclear export leads to autophagic flux and cell death. UVA-induced autophagosome-lysosomal dysfunction at the late phase causes cathepsin B inhibition, which might further amplify the autophagy cascade. On the other hand, EGFR transactivation is induced by UVA and functions to balance autophagy and reverse lysosomal function. Prior to UVA irradiation, pre-manipulation of AMPK activation would abrogate ROS-dependent downstream responses, and prevent autophagic death.

## Data Availability

Not applicable.

## Abbreviations

3-MA: 3-Methyladenine
γH2AX: H2A histone family member X (S139)
AKT: Ak strain transforming; protein kinase B
AMPK: AMP-activated protein kinase
ATG5: Autophagy related 5
ATP6V0D1: V-type proton ATPase subunit d1
CPD: Cyclobutane pyrimidine dimer
CTSB/ Cat B: Cathepsin B
DAPI: 4′,6-Diamidino-2-phenylindole
DDB2: DNA damage-binding protein 2
DRP-1: Dynamin-related protein 1
EGFR: Epidermal growth factor receptor
FITC: Fluorescein isothiocyanate
LAMP-1: Lysosomal-associated membrane protein 1
LC3: Microtubule-associated proteins 1A/1B light chain 3B
MMP: Mitochondria membrane potential
mTOR: Mammalian target of rapamycin
NAC: N-acetyl cysteine
OCR: Oxygen consumption rate
PAR: Poly (ADP-Ribose)
PARP-1: Poly [ADP-ribose] polymerase 1
PI: Propidium iodide
PI3K: Phosphoinositide 3-kinases
ROS: Reactive oxygen species
RPE: Retinal pigment epithelium
TFEB: Transcription factor EB
TGF-α: Transforming growth factor alpha
TKI: Tyrosine kinase inhibitor
TRPML1: Transient receptor potential cation channel, mucolipin subfamily, member 1
UVA/B/C: Ultraviolet wavelength A/B/C
XPC: Xeroderma pigmentosum C
